# A Multi-Level Decision Fusion Strategy for Condition Based Maintenance of Composite Structures

**DOI:** 10.3390/ma9090790

**Published:** 2016-09-21

**Authors:** Zahra Sharif Khodaei, M.H. Aliabadi

**Affiliations:** Department of Aeronautics, Imperial College London, South Kensington Campus, London SW7 2AZ, UK; m.h.aliabadi@imperial.ac.uk

**Keywords:** SHM, condition based monitoring, guided waves, electro-mechanical impedance, piezoelectric transducers, damage detection and characterization, maintenance strategy

## Abstract

In this work, a multi-level decision fusion strategy is proposed which weighs the Value of Information (VoI) against the intended functions of a Structural Health Monitoring (SHM) system. This paper presents a multi-level approach for three different maintenance strategies in which the performance of the SHM systems is evaluated against its intended functions. Level 1 diagnosis results in damage existence with minimum sensors covering a large area by finding the maximum energy difference for the guided waves propagating in pristine structure and the post-impact state; Level 2 diagnosis provides damage detection and approximate localization using an approach based on Electro-Mechanical Impedance (EMI) measures, while Level 3 characterizes damage (exact location and size) in addition to its detection by utilising a Weighted Energy Arrival Method (WEAM). The proposed multi-level strategy is verified and validated experimentally by detection of Barely Visible Impact Damage (BVID) on a curved composite fuselage panel.

## 1. Introduction

Structural Health Monitoring (SHM) technology is a relatively new concept which has emerged with advances in sensor technology and signal processing [1,2,3,4,5,6]. At present, the aircraft maintenance program includes the aircraft non destructive inspection (NDI) plan and implementation of a repair strategy. Based on the life interval of damage detection defined by the damage tolerance design, the maintenance is scheduled in predefined intervals (scheduled maintenance), which is attributed to high maintenance costs. The application of SHM to aircraft utilises integrated transducer networks to continuously acquire the state of the structure by real-time monitoring. The acquired data in combination with advanced signal processing techniques will lead to maintenance actions upon demand. The continuous monitoring of the aircraft can result in condition based maintenance (CBM), reducing the ground time and the maintenance costs significantly [7].

Based on the type of sensig and actuating technology, SHM systems can be divided into Passive and Active systems. Passive SHM systems use only sensors to provide impact detection [8,9,10], impact characterization [11,12,13] and damage detection [2]. By characterizing an impact event in terms of location and maximum contact force, the engineer can have an insight into whether the level of impact could have resulted in Barely Visible Impact Damage (BVID), or it is below a certain threshold and no immediate maintenance action is required. Another passive sensing technology for damage detection worth mentioning is the use of Fibre Optic sensors (FO). FO sensors can be embedded or surface mounted on the structure. There are numerous studies that have demonstrated the use of Fibre Bragg Gratings (FBGs) or FOs as strain measurement sensors either for damage detection [14,15] and/or load/strain monitoring [16,17,18].

Active sensing can provide information on detection, localization and identification of damage. There are numerous active sensing techniques based on different sensor technologies and signal processing methodologies. In terms of sensor technology, active sensing systems can be divided mainly into Piezoelectric (PZT) and hybrid (combination of FOs and PZTs) systems. For each of the mentioned SHM systems, there are various methodologies which have been developed, tested and validated over the past decade, mainly in laboratory environment. PZT transducers are used as actuators for exciting ultrasonic guided waves (UGW) as well as sensing the propagating wave due to their electro-mechanical coupling. They can be used for damage detection by actuating and sensing UGWs in the structure [19,20,21,22,23,24], vibration analysis [25,26,27] or by measuring the Electro-Mechanical Impedance response of the structure [28,29,30,31,32]. In addition, the advantages of utilising PZTs as actuators (i.e., low required power, low weight, multiple mode excitation) can be used together with FO sensors (high sensitivity to strain, low weight, temperature compensation) to design a hybrid system for damage detection [33,34]. Once an SHM system is selected, an important question is how many transducers are required and where their optimal locations are to reach the required probability of detection (POD) and reliability of the diagnostic system. There are different optimisation techniques developed for both passive [35,36,37,38] and active sensing [39,40,41].

The decision for any SHM system (for example [42]) to be considered as a diagnostic and prognistic system on board a real structure is driven by its additional cost, weight and the reliability of the decision making process. The benefit of installating a monitoring system can be assessd by quanitfying the Value of Information (VOI) [43]. The VOI concept can then be combined with structural reliability methods to efficiently compute the probability of system failure. Before any SHM system is designed for a specific POD and reliability, the engineer needs to define what is the intended function of the proposed diagnostic system. The intended function of any SHM system can be a selection or a combination of the following elementary functions: existence of damage, damage location, damage size and damage severity. Each intended function will require different number and location of transducers. Therefore, the VOI of the system will increase when the intended function becomes more complex. For example, if the output of the diagnostic system is existence of damage, a much smaller transducer network is required in comparison to locating the damage. Therefore, the VOI has a direct effect on assessing the benefits of an SHM system and the consequence maintenance plans. There are numerous publications and research projects dedicated to development and validation of different SHM systems for each intended function. However, very few of them have addressed the application of such diagnostic system to real complex structures such as aircraft sub-component and the required amount of data associated with them. Therefore, there remains the need for an approach which can be applicable to complex structures based on the intended function of the SHM system.

**Figure 1:**
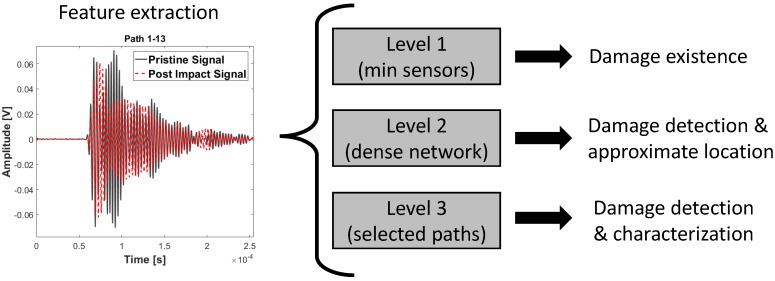
Multi-level diagnosis.

Rytter [44] has defined four levels on the damage assessment scale: Level I: Damage detection; Level II: Damage localization; Level III: Damage quantification and Level IV: Prognosis of the remaining service life. The aim of this paper is to investigate the effect of different intended functions on the accuracy and reliability of the SHM system and the extra cost associated with them in terms of the required data. The Level 1 diagnosis is based on the minimum required data for detecting existence of a damage. The proposed strategy is to have minimum number of transducers to cover large areas based on the acceptable attenuation levels, and to detect the existence of damage with high reliability. For the higher levels of the intended functions, a multi-level decision fusion is proposed based on UGW and EMI detection methodologies to reduce the amount and time required for the data acquisition on large complex structures. Level 2 diagnosis, in addition to damage detection, provides an approximate location of damage by identifying the maximum residual path, while Level 3 builds up on Level 2 diagnosis to result in accurate location and size of the damage. Damage quantification has not been addressed in this work, but this is in progress and will be presented in future work to complete the Level 3 diagnosis. The proposed multi-level strategy (Figure 1) is then tested on a curved composite fuselage panel for detecting BVID under the stiffener, which is one of the of most challenging damage detection scenarios for SHM techniques. All three levels of diagnosis have shown to provide reliable results based on the intended functions related to each level.

## 2. Diagnostics of a Fuselage Sub-Component

This section will present the developed damage detection and characterisation algorithm as part of a maintenance strategy for a fuselage sub-component (i.e., curved composite stiffened panel) based on a network of distributed PZT transducers and UGW acquisition. The experimental set-up is explained in Section 2.1. The first step is to evaluate the optimal parameters of the UGW acquisition system such as the actuation frequency, group velocity, attenuation profile based on the pristine state of the structure. After the sub-component is impacted, the post-processing of data are carried out for three different intended functions: Level 1 to determine the existence of damage; Level 2 to determine the existence of damage and its approximate location; and Level 3 to detect and characterize damage (exact location and size). Different methodologies are proposed for each level, as different cost is attributed to each level. The Level 1 diagnosis, as explained in Section 2.2, is based on the minimum required sensors to detect an existing damage in a large area of the structure; While Level 2, described in Section 2.3, proposes a detection and localization algorithm and Level 3 in Section 2.4 results in detection, localization and sizing methodology with minimum required transducers to minimize the cost/time for data acquisition and data handling. Although the proposed approach here is tested and validated for a fuselage panel, it is intended for large structures with multiple areas to be diagnosed, and hence the data acquisition and handling can be of large orders of magnitude. Level 3 diagnostics are referred to as damage characterization i.e., severity and type of damage in addition to its size. However, because the experimental panel was impacted only once to caused BVID, the severity of the damage could not be addressed in this work, and it is intended to be addressed in future works. Before the proposed methodologies are presented, the experimental set up is described in the following section.

### 2.1. Experimental Set-up

The fuselage panel that has been tested is a composite panel with 790 mm × 1150 mm dimensions and 1978 mm radius of curvature to the outer surface. The skin and omega hat stiffeners were made of T800/M21 uni-directional pre-pregs.

**Figure 2:**
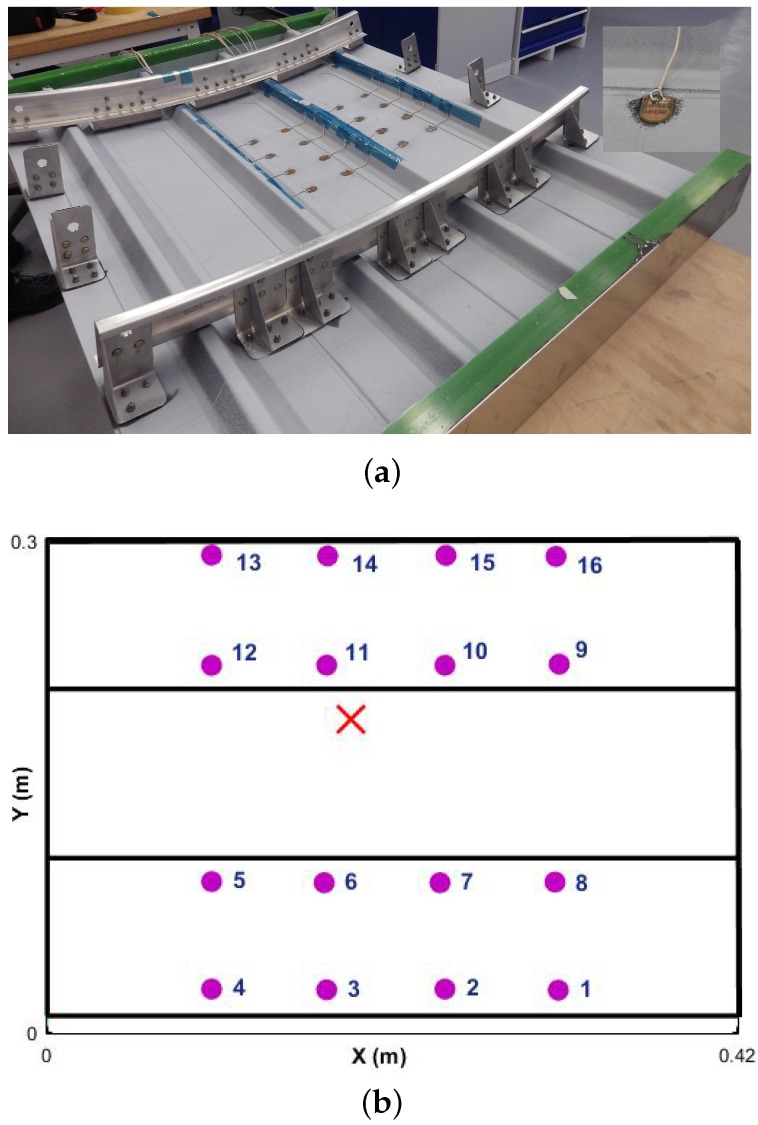
(**a**) Sensorized stiffened panel—DuraAct transducers; and (**b**) geometry of the sensor network.

The skin is 1.656 mm thick with (45/−45/90/0/90/0/90/−45/45) layup, whereas the stiffeners are 1.288 mm thick with (45/−45/0/90/0/−45/45) layup. A part of the the panel has been instrumented with 16 DuraAct PZT transducers (PI Ceramics, Karlsruhe, Germany) surface bonded, with cyanoacrylate Loctite 401 adhesive (Henkel, Düsseldorf, Germany), across two bays. The panel was over sensorized to collect as much data as possible and compare the reliability of the detection algorithm for different numbers and locations of transducers. The sensorized area is across two bays to aim for the most challenging case in terms of guided wave damage detection inspecting mid bay and under the stiffeners (see Figure 2).

**Figure 3:**
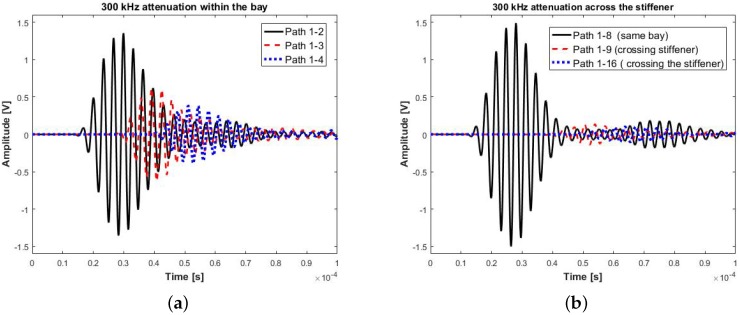
(**a**) Example of attenuated signal within a bay; and (**b**) example of attenuated signal across the stiffener.

The UGWs were recorded using a National Instrument platform with a PXIe single channel arbitrary voltage generator, PXI1 5105 digital oscilloscope and a Pickering 40-726A switching card with maximum output voltage of 12 Volts. To define the optimum parameters of the diagnosis system, the pristine panel has been excited with a five cycle Hanning tone-burst with central frequency range of 50 to 500 kHz in steps of 50 kHz. The signals were then recorded at 60 MS/s sampling rate for 0.001 s. Each transducer path was exited 10 times and signals were recorded, bandpass filtered and averaged to ensure repeatability and to minimize the background noise. The range of signals were then used to find the optimum amplitude, attenuation profile and group velocities of the stiffened panel. The geometry of the sensor network for the sensorized section of the panel is shown in Figure 2b. An example of the attenuation profile for 300 kHz excitation within the bay and across the stiffener is shown in two directions in Figure 3a,b. It is clearly shown that the attenuation across the stiffener is much more severe than within the bay. The maximum amplitude reduction within the bay is 55%, whereas, across the stiffener, this reduction is as high as 90%. Therefore, it is important to take this into consideration for optimising the number and location of transducers.

**Figure 4:**
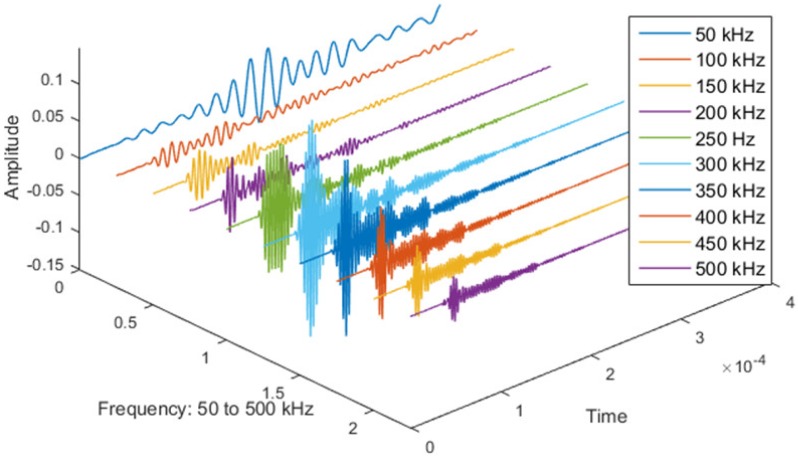
Max amplitude for the frequency sweep 50–500 kHz.

In order to minimize the effect of the attenuation, the frequency with the maximum amplitude response was chosen. The excitation frequency range 50–500 kHz where assessed, and the 300 kHz excitation was chosen as it generated the maximum response in the structure (see Figure 4).

Once all of the pristine data were recorded, the panel was subjected to 50 J impact under the stiffener from the outer skin to induce BVID, i.e., skin/stringer debonding, which represents the most severe case of un-detected damage for a stiffened panel. In addition, detecting skin/stringer debonding is one of the most challenging damage scenarios for the SHM system to detect due to the propagation profile of the guided waves, such as changes in the thickness, additional reflections due to stiffener edges, and attenuation. The location of damage has been indicated by a red X mark in Figure 2b. An INSTRON CEAST 9350 drop tower with a 20 mm radius hemispherical impactor was used. Both finite element (FE) simulation and experimental results showed that there was a 10% reduction in the compressive strength of the damaged panel [45]. The C-scan results confirmed a debonded area that was not visible to the naked eye. After the impact, the UGW data were recorded again for damage detection.

### 2.2. Level 1 Diagnosis: Damage Existence

**Figure 5:**
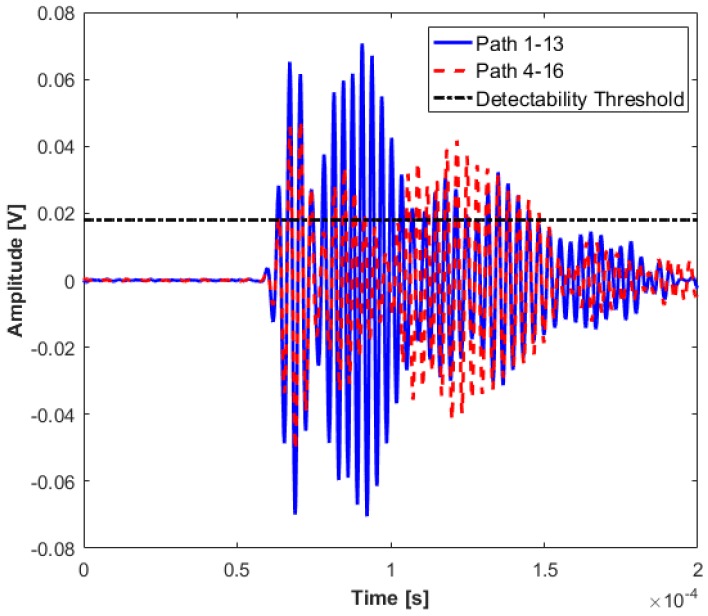
Attenuated signals for Level 1 detection.

The aim of Level 1 diagnosis is to inform the maintenance engineer of the existence of any damage with minimum cost to the acquisition system. The proposed algorithm for Level 1 aims to cover the maximum area of the structure with the minimum number of transducers. In this case, a transducer network {1 4 13 16} has been chosen for detecting existence of damage. Since the network crosses the stiffener, the first step is to check if the attenuation of the signals is acceptable for detecting any damage inside the sensor network. In theory, reciprocity of the system must hold, which means the signals from path 1-2 must be identical to path 2-1. However, in real structures, there are tolerances with respect to installation of the transducers, which results in small variation in identical paths. These small perturbations have been measured for the total sensor network, and the detectability threshold was set to be 20% above this level to ensure that the attenuation of the signals crossing the stiffener, on its longest paths (i.e., 1-13 and 4-16), does not fall below this limit. As can be seen from Figure 5, the amplitudes of the signals for the two paths mentioned above are significantly higher than the detectability threshold, and thus the sensor network can be used for Level 1 detection.

**Figure 6:**
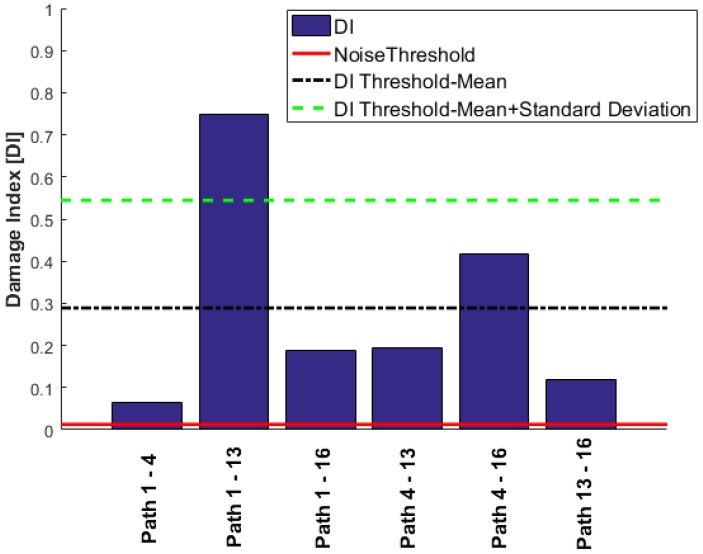
Level 1 detection with only four corner transducers.

Level 1 detection is based on UGWs by comparing the baseline with the current state of the structure, i.e., post impact in this experiment. A damage index (DI) measure is proposed to represent existence of damage where 0 means no damage and 1 indicates damage. The DI proposed for Level 1 is introduced as the maximum energy envelope of the residual signals for each path, where the residual ($R_{ij}\left(t\right)$) is defined as the difference in the energy envelope between the pristine state ($S_{ij}\left(t\right)$) and the current state ($P_{ij}\left(t\right)$) for each path ij. The energy envelops difference between the pristine state and the current state is given by:

(1) $$E\left(R_{ij}\right)=\left|R_{ij}-iH\left(R_{ij}\right)\right|,$$

where H(R_ij) is the Hilbert transform of the residual signal. Then, the DI for each path *ij* is defined as the $max(E\left(R_{ij}\right)$. Before running the proposed diagnostic algorithm, a threshold value must be defined to indicate damage. To define a damage threshold, usually a large library of baseline measures is required to carry out a statistical analysis to separate the environmental effects from the damage reflected signals. For this experiment, each measurement was repeated 10 times and the reversibility of each path was also used to define a noise threshold at the first stage. The DIs for all the paths of the sensor network {1 4 13 16} are presented in Figure 6, and the noise threshold is indicated by the red solid line. The values above the noise threshold indicate changes in the signal but not necessarily due to existence of damage. These changes could be attributed to factors such as boundary conditions, loading, temperature, hardware. The second stage is to define a damage threshold to reliably detect the existence of damage. For this reason, the “DI Threshold-Mean” value was identified as the average DI values from all the paths. Any value above this threshold will indicate damage in the close proximity of the path. The impact location is directly over path 1-13 as indicated in Figure 2b and very close to path 4-16. It is clearly seen that the DI values for these two paths are above the set threshold for damage existence. Another threshold has been defined to indicate a proximal location of damage. This threshold is set as the mean value of all the DIs + the standard deviation and is shown by the dashed green line in Figure 6. The DI plots clearly indicate damage existence and also approximate location of damage on the path 1-13 as Level 1 detection using only four transducers in the corners to cover a large area within the transducer network.

### 2.3. Level 2 Diagnosis: Damage Existence and Location

The Level 1 diagnosis resulted in existence of damage and detecting its proximal location, i.e., path 1-13. However, if these results were to be used for a maintenance action, the engineer would then have to monitor a large area of mid-bays, and, under the stiffeners with an NDI technique, localize the damage and take consequent repair actions. If the damage is located in mid-bay, the consequences of not detecting it will not be as severe as debonding of the skin/stringer. Therefore, for some areas, not only the presence of damage is of high importance but to be able to locate it with high precision as well.

There are numerous probability based imaging algorithms that result in damage detection and identification. The methodology which is adopted in this work for localizing and possibly sizing the damage is based on the weighted energy arrival method (WEAM) presented in [22]. A brief explanation of the method will be given in this section; however, for more detailed overview, please refer to [22]. The WEAM algorithm is based on a probability damage index for every point in the structure and an imaging algorithm to plot the points with highest probability of damage existence. The damage index at every point in the structure from each transducer path is measured by:

(2) $$DI\left(x,y\right)=\frac{1}{N}\sum_{i=1}^{N}\sum_{j=1}^{N}E\left(R_{ij}\left(t_{ij}\left(x,y\right)\right)\right).w_{ij}\left(\sigma ,\nu \right),$$

where $E(R_{ij}\left(t_{ij}\left(x,y\right)\right))$ is the energy envelope of the residual for the path *ij* calculated from Equation (1), at the location *x,y*, and time *t*. $w_{ij}\left(\sigma ,\nu \right)$ is a window function with log-normal distribution centred at the first peak of the residual signal to reduce the effect of boundary reflection and mode superposition.

**Figure 7:**
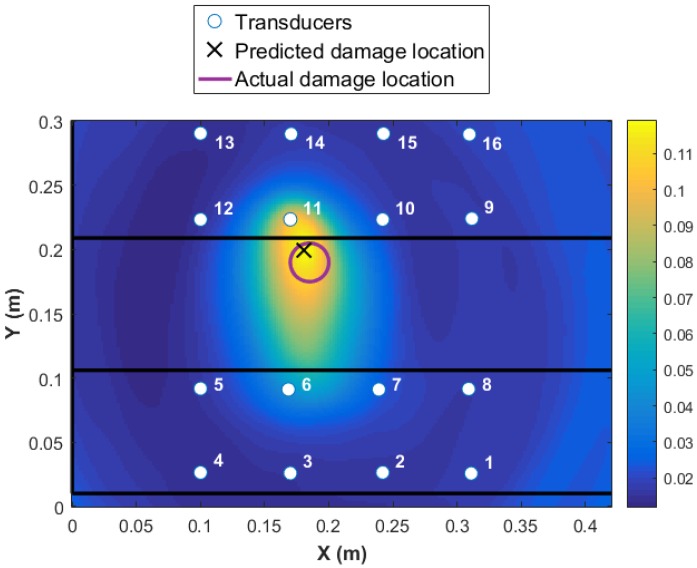
Damage detection and identification with all 16 transducers using Weighted Energy Arrival Method.

**Figure 8:**
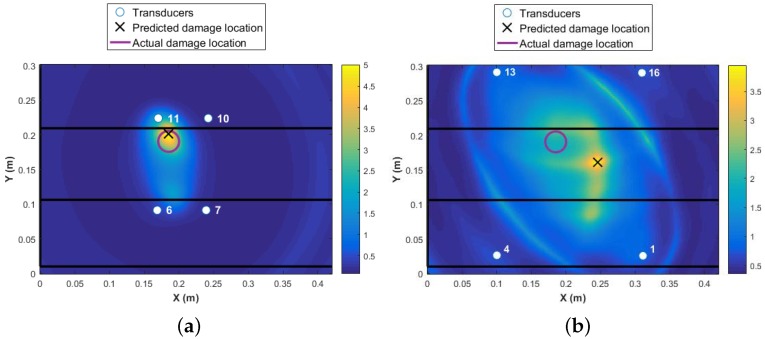
Damage detection and identification: (**a**) four transducers close to damage; (**b**) four most corner transducers.

**Figure 9:**
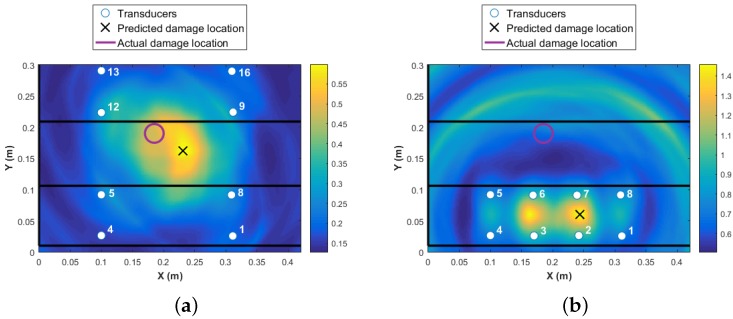
Damage detection and identification with different sensor network: (**a**) six distributed transducers; (**b**) eight transducers in one bay.

A threshold limit is implemented based on the noise level in the data. At the first step, the data collected from all 16 transducers are used for damage detection and identification as presented in Figure 7. The colormap in the plot shows the DI values at every point in the structure, with the highest value representing the area with the highest probability of damage existence. The higher the DI value, the more reliable is the diagnosis, i.e., significantly above noise threshold and changes due to operational and environmental conditions. The red circle in Figure 7 represents the real damage size and area, and the black cross represents the predicted location with the highlighted size. It is shown that the damage is detected with high accuracy. However, to use 16 transducers (240 paths) is expensive in terms of time of the acquisition and data-processing. Each path took about four minutes. In addition, the maximum value of the DI is about 0.12 as presented in Figure 7. This value is obtained by fusing the residuals from all transducer pairs using an appropriate fusion algorithm, Equation (2). Therefore, the contribution of the paths that are far from the damage is negative to the overall detection probability. Ideally, by using the minimum number of paths that are in the proximity of damage, a much higher probability of locating the damage will be reached. For example, if the transducer network {6 7 10 11} is used, the maximum value of DI will increase to five, which is 41 times higher than using 16 transducers. This shows that the reliability of detection is much higher for the four-transducer network because the DI values are far from the noise levels and the damage localization and size is much more accurate (see Figure 8a). In contrast, if a different combination of four transducers were used with paths that pass close to the damage but further apart from each other, for example, the same sensor network as in Level 1 diagnostics {1 4 13 16}, even though the magnitude of DI remains high for detecting damage reliably, the localization is poor, as presented in Figure 8b. To demonstrate the sensitivity of the transducer number and location of the damage detection and localization, results from two different networks are presented in Figure 9.

It is clear from the above results that if the intended function of the SHM system is to locate and characterise the damage in addition to its existence, then a dense network of transducers is required. In contrast, for detecting damage existence, the DI values from four transducers provide values that are significantly above the noise levels. For damage localization to result in accurate damage location, however, not all the transducers are required to be used in the second level diagnosis (see Figure 8). The question which is now raised is how the user can determine which transducers are to be used in the second- and third-level detection without the prior knowledge of where the impact might have occurred.

#### 2.3.1. A Multi-Level Solution

An important aspect of any proposed diagnostic system is its reliability and probability of detection. This means that the probability of false alarm or misdetection must be minimal. One factor that contributes to false alarm is the failure of the interrogation system, and, in particular, the sensors. PZT transducers are ceramic and thus brittle. Therefore, for real applications, they must be protected—for example, DuraAct transducers (PI Ceramics, Karlsruhe, Germany) have a protection layer. In addition, their bonding to the structure is of high importance, since any deterioration in the bonding quality or connections will result in a change in signal amplitudes and can be identified as false alarms. Therefore, a self-diagnostic scheme must be in place to check the quality of the SHM system (sensors and connections) after installation and prior to its being interrogated for diagnosis to increase the reliability of the decision making process. The self-diagnostic methodology proposed here is based on the EMI measures of the system to detect any faulty sensors [30].

**Figure 10:**
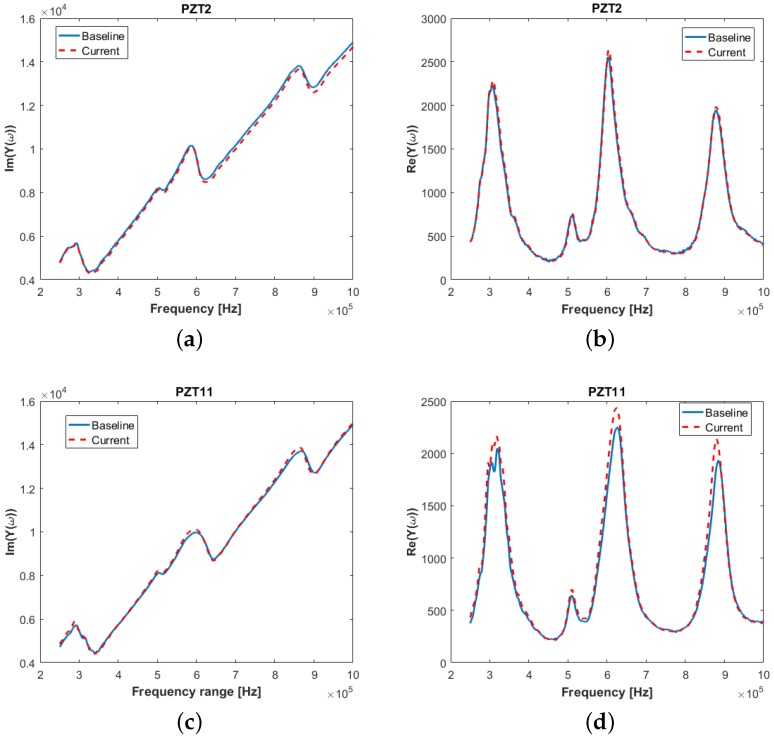
Electrical admittance measurement from Piezoelectric sensors (**a**) imaginary part of admittance—PZT2; (**b**) real part of admittance—PZT2; (**c**) Imaginary part of admittance—PZT11; and (**d**) real part of admittance—PZT11.

EMI technique can be used to measure the local dynamical response of a structure at the location of the PZT transducers due to their electro-mechanical coupling. A harmonic voltage is applied to terminals of a PZT transducer and the current propagating through the surface is measured. This is a very attractive method, as each transducer is excited and sensed individually, which simplifies the data acquisition and post-processing. It also reduces the required acquisition time in comparison to UGW acquisitions. When a non-linearity such as damage is present in the vicinity of the transducer, it will change the dynamic response of the structure, and, by comparing its response to a baseline measure, damage can be detected. Damage can either be damage in the transducers (failed sensors, detached sensors) or damage to the structure (delamination, debonding, softening). The electromechanical admittance *Y* is defined as the ration between the current *I* and the excitation voltage *V*. The complex electro-mechanical admittance is expressed as:

(3) Y=ω(Re(Q)Im(V)-Im(Q)Re(V)Re(V2)+Im(V2)+jω(Re(Q)Re(V)-Im(Q)Im(V)Re(V2)+Im(V2),

where *ω* is the excitation frequency and *Q* is the electric charge. The impedance is the inverse of the admittance $Z=Y^{-1}$. The difference in the admittance measured in a healthy structure, and a faulty one is then used to detect damage. This difference in the EMI spectrum measured over a range of frequencies is related to a damage metric value when above a defined threshold. Figure 10 illustrates the changes in the real and imaginary part of admittance measured for a frequency range of 250 to 1 MHz, for two different sensors attached to the composite stiffened panel. PZT2 is located far from the impact damage, whereas PZT11 is very close to the impact site. It can be seen that, while the slope of the imaginary part of the admittance changes slightly (within noise level), the peaks of the real part of admittance changes noticeably for PZT11 when damage is present. This also confirms the findings of other researchers that the slope of the admittance is related to the diagnosis of the sensor alone (i.e., sensor integrity and bonding quality) [46], and the real part of admittance can be used for detecting damage in the structure in the vicinity of the transducer [47].

**Figure 11:**
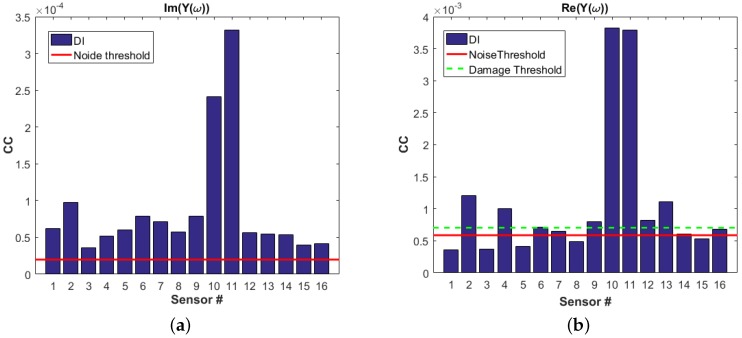
Electro-mechanical Impedance damage metric based on: (**a**) imaginary part of admittance; and (**b**) real part of admittance.

In this work, the damage metric for the EMI approach is defined as the piecewise linear correlation coefficient CC between the pristine data and the current data. The noise threshold is measured from the pristine data by averaging the transducer responses measured over several days. The damage threshold then is defined as 20% above the noise threshold. Figure 11 depicts the DIs in a bar plot for both imaginary and real part of admittance for all transducers and the respective noise thresholds. It is shown that, although both damage metrics show DI values above the noise threshold, CC coefficients measured from the real part of admittance are more conclusive with the damage existence in the structure. The DI values for PZT10 and 11 are the highest indicating damage in the vicinity of transducers 10 and 11, which can be validated with the location of the BVID.

### 2.4. Level 3 Diagnosis: Damage Characterization

By processing the DI values from Level 2 diagnosis, the maintenance engineer can confirm the existence of damage and its proximity to transducers 10 and 11. The exact location, size or type of damage cannot be defined. However, this information can be used in a multi-level diagnostic strategy to reduce the amount of data acquisition and increase the reliability of the decision making. The Level 2 decision making resulted in existence of damage close to transducers 10 and 11. From Figure 8, it was concluded that, when applying the WEAM algorithm, to ensure the correct damage characterization, the number of direct paths passing through the damage must be maximized while the paths that are far from damage should be minimized (to increase the reliability). As mentioned before, to cover the whole structure, a dense network of transducers is required. However, for a detailed damage detection and characterization with high reliability, not all of the transducer paths are required to be used. The paths that are far from damage have no positive contributions to the detection algorithm and will have a negative impact on the output of the data fusion. Therefore, Level 3 diagnosis can be built on the decisions from Level 2 to provide a robust damage identification with minimal cost. Level 3 aims to characterize the damage, i.e., its exact location, type and severity. In this work, only the exact location and size has been addressed in Level 3, and the authors are progressing with characterizing the type and severity of damage in future works.

**Figure 12:**
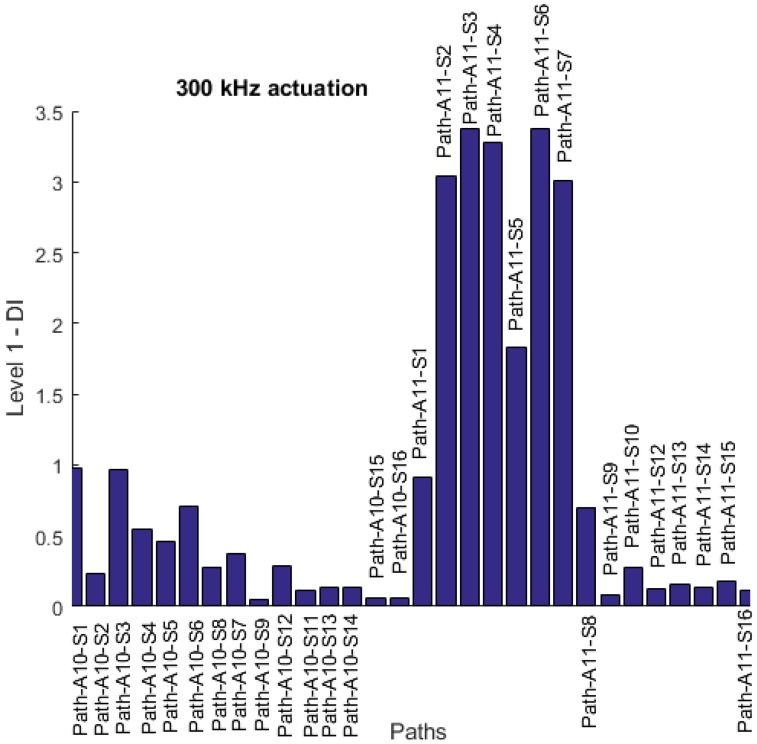
Maximum residual path (MRP) based on Level 1 DIs for actuators 10 and 11.

The minimum number of transducers that is required for a delay and sum damage detection algorithm based on triangulation is three. Therefore, from the EMI measures, transducers 10 and 11 are chosen as the two transducers that can be used for a reliable damage localization in Level 3 diagnosis. The reason why these two traducers are chosen is that their DI values are significantly higher than the rest of the transducers and the damage threshold, to ensure reliable selection. There is at least one more transducer required for triangulation. However, the choice of this transducer will have a direct effect on the accuracy of the result. The WEAM will have the best result when the damage is inside the transducer network. The selection criteria proposed in this work is to use the maximum residual path (MRP) to choose a transducer that has the maximum effect of damage presence on its paths. To choose the last transducer required for damage localization, transducers 10 and 11 are used as actuators and using Level 1 damage detection; as described in Section 2.2, the DI values for all the paths are plotted in Figure 12. It is observed that maximum residual paths are 11-3 and 11-6. This means that the probability of damage being on these direct paths is the highest. To select between the transducers 3 and 6, the path lengths 11-6 and 11-3 are compared. It can be seen that the path 11-6 is shorter. Therefore, transducer 6 is chosen as the one closer to the damage site. This means that the damage is inside the transducer network {10 11 6}, and the reliability of the damage detection and identification is increased.

The next step of the multi-level diagnosis is to run the WEAM algorithm using the transducer network {10 11 6}.

**Figure 13:**
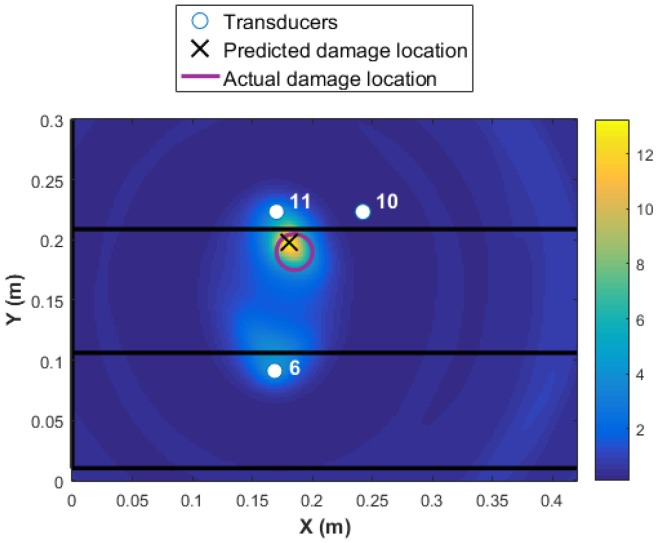
Level 2 detection based on MRP transducer network 1.

**Figure 14:**
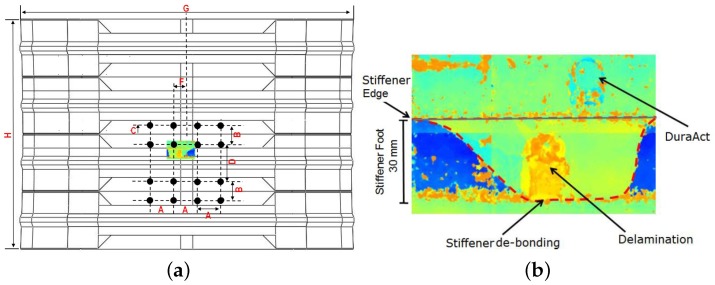
Fuselage panel C-scan image after impact. (**a**) Full scale; (**b**) Enlarged scale.

The results of Level 3 detection based on the WEAM for the transducer network with MRPs is shown in Figure 13. It can be seen that not only the accuracy of the detection is increased when using the transducer network based on MRP, but the reliability of detection (increased DI magnitudes) and the accuracy of the damage size has improved significantly. The C-scan results of the stiffened panel post impact is presented in Figure 14, which validates the damage detection and identification results from the multi-level diagnostic method.

**Figure 15:**
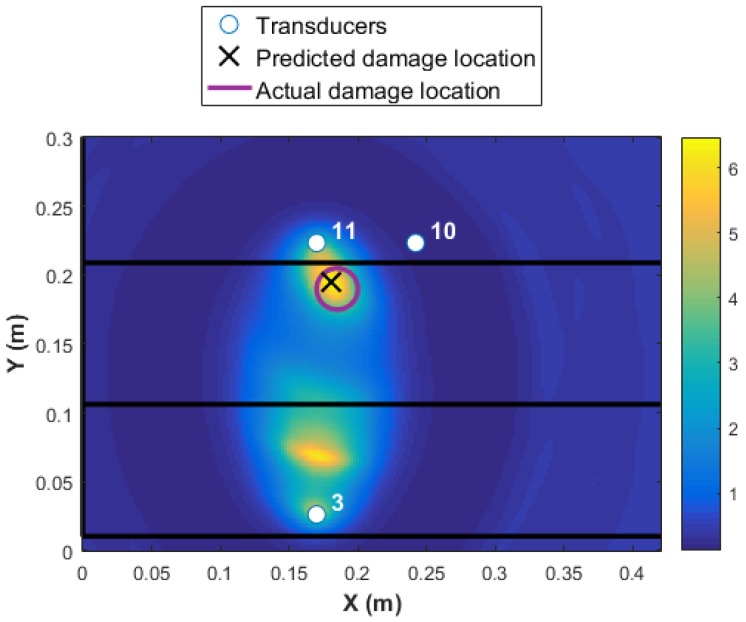
Level 2 detection based on MRP transducer network 2.

Even if the transducer network {10 11 3} is used for Level 3 damage detection and identification, damage is detected and localized accurately as presented in Figure 15. However, the reliability is not as high as the MRP transducer network 1 as the maximum amplitude of the DI reduced from 12 to 6, and there is an additional area with a high probability of damage existence close to transducer 3. It is worth highlighting that a network with only three transducers are used in this case, which reduces the signal pre and post-processing significantly (only six transducer paths).

## 3. Conclusions

In this work, a multi-level diagnostic strategy has been proposed for three different intended functions of SHM: Level 1—damage existence; Level 2—damage detection and approximate location; and Level 3—damage detection and identification. The advantage of the proposed strategy is that it is readily applicable and upscalable to large and complex structures. The proposed algorithm has been validated on a curved composite fuselage panel impacted to cause BVID. Using Level 1 detection, it was shown that, by using four transducers, the damage was accurately predicted above a defined threshold and that the maximum residual corresponded to the direct path going through damage. However, the exact location of damage could not be detected. Level 2 diagnosis based on EMI measures showed that damage is clearly detected and also an approximate location can be identified close to the transducers with the highest change in their admittance response. These transducers were then used as actuators in the third level of diagnostics based on UGW acquisitions, while the rest of the transducers were recording the propagating wave. The DI strategy proposed for Level 1 was then used to define the MRPs and consequently find the three transducers with the highest residuals in their path and use this network for damage detection and characterization based on WEAM. The results show the importance of the number and location of the transducers on the accuracy and reliability of the damage detection algorithm. Therefore, the proposed algorithm has a higher accuracy for damage localization and sizing when it uses the multi-level decision fusion in comparison to using all the available transducers on the structure. It shows that not always increasing the number of transducers will result in a more accurate detection and characterisation. Consequently, for each intended function, the VoI must be weighed against additional weight and cost that the SHM imposes on the structures and the benefits of a CBM analysed and an appropriate maintenance schedule is planned accordingly.

The advantage of the proposed method is that it is readily scalable for large complex structures and reduces the risk of misdetection and false alarm by fusing information across different levels of diagnosis. In addition, it offers different algorithms for each intended function to increase the accuracy and reliability of each function based on their output, and optimise the amount of the required data acquisition, handling and processing. The validity of the proposed methodology under real load conditions is being assessed and will be reported in future publications. Some validations are available for temperature compensation [23].
